# Current perspectives on the immunosuppressive tumor microenvironment in hepatocellular carcinoma: challenges and opportunities

**DOI:** 10.1186/s12943-019-1047-6

**Published:** 2019-08-29

**Authors:** Chen Lu, Dawei Rong, Betty Zhang, Wubin Zheng, Xuehao Wang, Ziyi Chen, Weiwei Tang

**Affiliations:** 1 Department of General Surgery, Nanjing First Hospital, Nanjing Medical University, Nanjing, Jiangsu China; 2 Hepatobiliary/Liver Transplantation Center, The First Affiliated Hospital of Nanjing Medical University, Nanjing, Jiangsu China; 3 Key Laboratory of Living Donor Transplantation, Chinese Academy of Medical Sciences, Nanjing, Jiangsu China; 4 Michael G. DeGroote School of Medicine, McMaster University, Hamilton, Ontario Canada; 5 Department of General Surgery, Zhongda Hospital, Medical School, Southeast University, Nanjing, Jiangsu China

**Keywords:** Hepatocellular carcinoma, Macrophages, Myeloid suppressor cells, Immunotherapy

## Abstract

Incidence of hepatocellular carcinoma (HCC) is on the rise due to the prevalence of chronic hepatitis and cirrhosis. Although there are surgical and chemotherapy treatment avenues the mortality rate of HCC remains high. Immunotherapy is currently the new frontier of cancer treatment and the immunobiology of HCC is emerging as an area for further exploration. The tumor microenvironment coexists and interacts with various immune cells to sustain the growth of HCC. Thus, immunosuppressive cells play an important role in the anti-tumor immune response. This review will discuss the current concepts of immunosuppressive cells, including tumor-associated macrophages, marrow-derived suppressor cells, tumor-associated neutrophils, cancer-associated fibroblasts, and regulatory T cell interactions to actively promote tumorigenesis. It further elaborates on current treatment modalities and future areas of exploration.

## Introduction

Hepatocellular carcinoma (HCC) is the second leading cause of cancer-related deaths worldwide, with approximately 800,000 cases per year [1]. Most cases appear in the context of cirrhosis and are most often associated with chronic hepatitis B and hepatitis C virus infection. In the case of localized HCC, surgical resection, liver transplantation, and tumor ablation are potential cures. Advancements such as laparoscopic liver resection and living donor transplantation continue to develop and influence treatment options [2, 3]. For patients with locally advanced disease, interventional techniques such as transarterial chemoembolization or transradial radioembolization can provide disease control or lead to tumor regression and hypertrophy in future liver remnants [4, 5]. Systemic sorafenib has been shown to be effective in patients with severe cirrhosis who are not suitable for liver-directed therapy and patients with metastatic HCC who have slow disease progression. However, it only exerts a weak therapeutic effect [6, 7]. Therefore, a novel treatment strategy with different mechanisms from those of conventional treatments is needed to improve the prognosis of HCC.

Since the approval of cytotoxic T lymphocyte-associated protein 4 (CTLA-4) and programmed cell death protein 1 (PD-1) inhibitors for the treatment of melanoma, immunotherapy has emerged as a potential alternative treatment option in clinical practice. Get widespread attention. Malignant tumors [8]. HCC is an inflammation-driven disease with potentially chronic liver inflammation and cirrhosis, and a quarter of HCC cases express markers of inflammatory response. However, these tumors also have fewer chromosomal aberrations, suggesting a combination of immunological interventions may be more effective with conventional treatment of this disease [9]. Major immunotherapeutic strategies for HCC can be classified into five categories: adoptive cell therapies; cytokines; vaccines; immune checkpoint inhibitors; and oncolytic viruses. A phase II clinical trial using tremelimumab included 21 patients with advanced HCC, with partial response rates and disease control rates of 17.6 and 76.4%, respectively [10]. PD-1 is expressed on B cells, T cells, natural killer (NK) cells, and dendritic cells (DCs) [11]. The PD-1 monoclonal antibody (mAb) blocks receptor binding of PD-L1 and PD-L2 to activate immune cells [12]. The researchers found that the PD-1 inhibitor nivolumab activates a sustained tumor-specific immune response and that side effects are controllable [13]. Treatment with PD-1 and CTLA-4 can stimulate T cell activation to enhance tumor eradication.

In the tumor microenvironment, non-malignant cells can help tumor cells to proliferate, invade and metastasize. The immunosuppressive features of tumor lesions participate not only as one of the major players inducing cancer progression but also a big challenge for effective immunotherapy. It has been found that immunosuppression associated with chronic inflammatory factors, such as growth factors, cytokines, and chemokines is generated by stroma and tumor cells [14, 15]. Multiple immune cells coexist and interact in a complex series of pathways that ultimately lead to tumor carcinogenesis. In the review, we will document some immunosuppressive cells, including tumor-associated macrophages (TAMs), marrow-derived suppressor cells (MDSCs), tumor-associated neutrophils (TANs), cancer-associated fibroblasts (CAFs) and regulatory T cells (Tregs) and their roles in cancer formation, which can lead to HCC.

### Tumor-associated macrophages

**Fig. 1:**
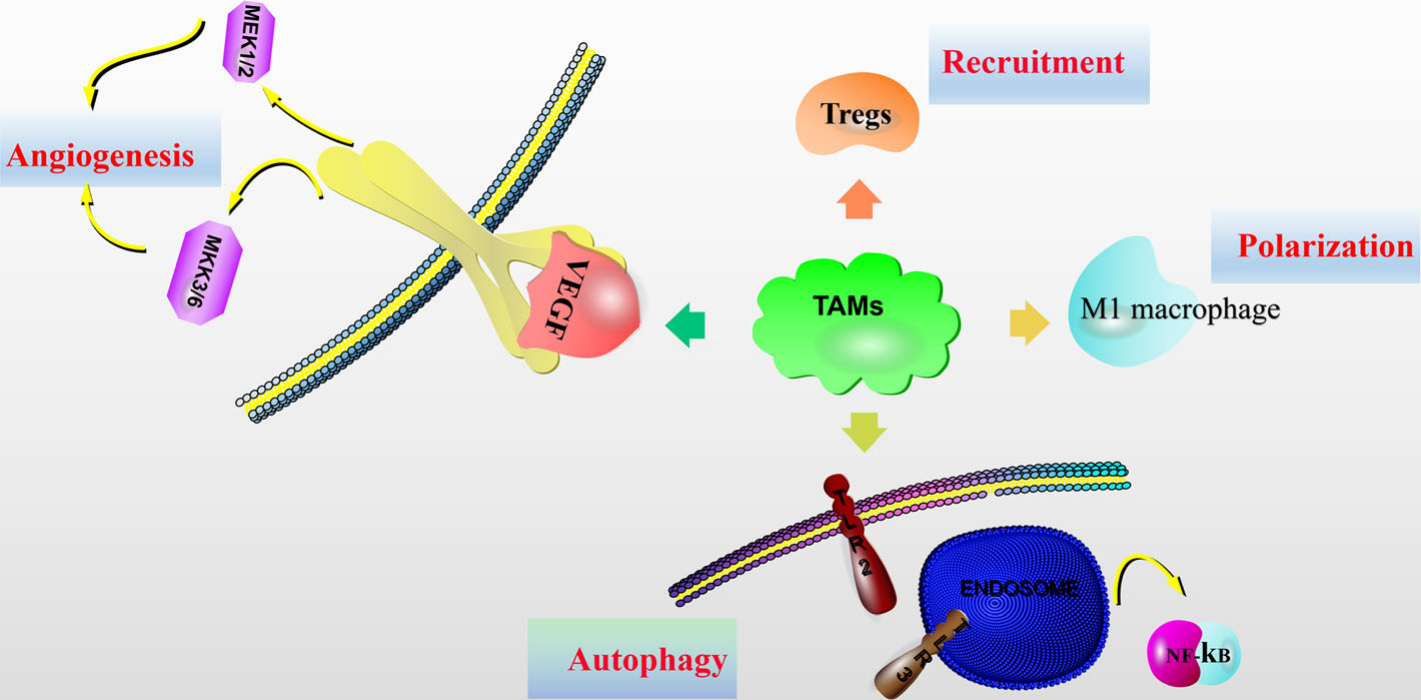
Macrophage targeting strategy in HCC therapy. Preclinical studies have identified key pathways that regulate the recruitment, polarization, survival, and autophagy of TAMs during tumor progression. In addition, inhibition of macrophage-derived VEGF can inhibit tumor angiogenesis and progression. Targeting key receptors or signaling proteins can inhibit these macrophage properties and inhibit tumor progression. These molecular targets form the basis of several therapeutic HCC strategies currently being clinically developed to promote an effective anti-tumor immune response. Abbreviations: TAM, tumor-associated macrophages; Treg cells: regulatory T cells;VEGF, vascular endothelial growth factor

TAMs inhibit anti-tumor immunity and promote tumor progression by expressing cytokines and chemokines. Preclinical studies have identified key pathways for TAMs recruitment, polarization and metabolism during tumor progression, and new therapies for these pathways can indirectly stimulate cytotoxic T-cell activation and recruitment [16–18]. Clinical trials of therapeutic agents currently promoting phagocytosis or inhibiting the survival, proliferation, transport or polarization of TAMs have shown improvement of cancer outcomes (Fig. 1).

TAMs are significant components of the microenvironment in HCC and associated with a poor prognosis of HCC patients. TAMs expression and density have been assessed by immunohistochemical staining from 253 HCC patients and results showed that CD68^+^ TAMs were not associated with clinicopathologic features and prognosis in HCC. However, low presence of CD86^+^ TAMs and high presence of CD206^+^ TAMs were significantly associated with invasive tumor phenotypes and with poorer overall survival (OS) as well as reduced time to recurrence [19]. TAMs develop from monocytes to functional macrophages and acquire various immunosuppressive functions at each stage of its differentiation to maintain the tumor microenvironment [20]. Repolarization of TAMs towards an antitumor phenotype was one approach to contributing to tumor regression [21]. Yang Y et al. first demonstrated that tumor cell-derived Wnt ligand stimulates M2 to transduce the polarization of TAMs via classical Wnt/β-catenin signaling, which results in immunosuppression in HCC [22]. Therefore, blocking Wnt secretion in tumor cells and/or activation of Wnt/β-catenin signaling in TAMs may be a potential strategy for future HCC treatment.

Previous literature has demonstrated that TAMs can produce a variety of chemokines, such as CCL17, CCL18 and CCL22, which attract Tregs cells to cancer sites, thereby impeding cytotoxic T cell activation [23, 24]. This may lead to a positive feedback loop between TAMs and Tregs, providing a new dimension to the immunosuppressive effects of cancer. TAMs in HCC can also attract Tregs to regulate the tumor microenvironment. This phenomenon was found by Zhou J that the intratumoral distribution of FoxP3^+^ Tregs were associated with high density TAMs. The anti-interleukin-10 (IL-10) antibody partially blocked this increase, suggesting that TAMs may trigger an increase in the population of FoxP3^+^ Treg cells in the tumor, thereby promoting the progression of HCC [25]. Mechanistically, Wu Q et al. recently confirmed that TREM-1^+^ TAMs can respond to hypoxia and tumor metabolites via the ERK/NF-κβ pathway leading to accumulation of CCR6^+^ Foxp3^+^ Tregs [26]. This study highlights hypoxia-induced tumor immunosuppression by TREM-1^+^ TAMs that attracts CCR6 ^+^ Foxp3 ^+^ Tregs, and TREM-1^+^ TAMs confers HCC resistance to Programmed cell death 1 ligand 1 (PD-L1) treatment.

TAMs are able to produce angiogenic factors, such as vascular endothelial growth factor (VEGF), platelet-derived growth factor and transforming growth factor β. TAMS also induce angiogenesis by expressing matrix metalloproteinase (MMPs) [27–29]. Angiogenesis inhibition therapy has recently become a promising therapeutic strategy for HCC. Lipid-based nanoparticles (NP) targeting CXCR4 were developed to deliver VEGF siRNA specifically to HCC as an anti-angiogenic substance. The researchers demonstrated that AMD-modified NPs (AMD-NPs) can effectively transfer VEGF siRNAs into HCC and down-regulate VEGF expression in vitro and in vivo. Although SDF1α/CXCR4 axis is upregulation in hypoxia induction after anti-angiogenesis therapy, AMD-NPs combined with conventional sorafenib therapy or VEGF siRNA inhibition of CXCR4 can prevent the infiltration of TAMs [30]. However, has there been any association between TAMs and VEGF in HCC? One study has investigated the role of macrophages in HCC progression under sorafenib treatment and explored whether combination of drugs that deplete macrophages improved the antitumor effect of sorafenib. Results showed that although sorafenib significantly inhibited tumor growth and lung metastasis, it induced a significant increase in peripheral recruitment and intratumoral infiltration of F4/80- and CD11b-positive cells, which causes an increase of VEGF. Depletion of macrophages by clodrolip or zoledronic acid (ZA) in combination with sorafenib significantly inhibited tumor progression, tumor angiogenesis, and lung metastasis [31]. These results indicate macrophages may have an important role in tumor progression under sorafenib treatment.

Preclinical studies have established that autophagy may play a role in the manipulation of TAMs function and tumor-related immunity. Chang CP et al. recently found that toll-like receptor 2 (TLR2)-associated ligands derived from HCC can stimulate the differentiation of M2 macrophages by selectively autophagy to control the homeostasis of NF-κβ RELA/p65 protein. TLR2 signaling induces NF-κβ RELA cytoplasmic ubiquitination and leads to degradation by SQSTM1/p62-mediated autophagy. Inhibition of autophagy will save NF-κβ activity and shape the phenotype of HCC polarized M2 macrophages [32]. The findings reveal a new role for autophagy in TAMs, which controls cellular function. Previous studies reported that Abies georgei’s natural product, named 747, is associated with xenophenol structure and exhibits sensitivity and selectivity as a CCR2 antagonist. In animals, 747 increases the number of CD8^+^ T cells in tumors by blocking tumor-infiltrating macrophage-mediated immunosuppression and inhibits in situ and subcutaneous tumor growth in a CD8^+^ T cell-dependent manner. In addition, 747 enhances the therapeutic effect of low-dose sorafenib without significant toxicity [33]. As shown in Table 1, treatment of TAMs targets in HCC has been documented and could be a new approach for treating HCC [34–38].

**Table 1:** A Summary of Molecule Target Therapies in HCC

Molecule	Target cell	Therapeutic setting	Major effects	Reference
CCR2	TAMs	CCR2 antagonist	Inhibits the recruitment of inflammatory monocytes, infiltration and M2-polarisation of TAMs	[34]
CCR2	TAMs	Anti-CCR2	Promotes epithelial‑to‑mesenchymal transition by upregulating matrix metalloproteinase‑2	[35]
CCR2	TAMs	CCR2 monoclonal antibody	Inhibits recruitment of monocytes	[36]
CSF-1	TAMs	CSF-1 receptor antagonist	Reprograms polarization of TAMs	[37]
IL-6	TAMs	Anti-IL-6	Blocks downstream effect of TAM products	[38]
IL-6	MDSCs	Anti-IL-6	IL-6 expression levels strongly correlate with an MDSC phenotype and chemotherapy response in HCC patients	[44]
chemokine (C-C motif) ligand 26	MDSCs	Blockade of chemokine (C-C motif) ligand 26	Knockdown of chemokine (C-C motif) ligand 26 in cancer cells profoundly reduces MDSC recruitment, angiogenesis, and tumor growth	[45]
SSAO	MDSCs	SSAO inhibitors	May have an anti-tumor effect on HCC by inhibiting recruitment of CD11b^+^ and Gr-1^+^ cells and hindering angiogenesis	[46]
STAT3	MDSCs	Anti-STAT3	Inhibiting STAT3 can enhance the clinical efficacy of CAR-T cells in LM through modulation of L-MDSC	[47]
CCRK	MDSCs	Anti-CCRK	Hepatic CCRK induction in transgenic mice stimulates mTORC1-dependent G-csf expression to enhance polymorphonuclear MDSCs recruitment and tumorigenicity in HCC	[48]
CCL9/CCR1	MDSCs	Blockade of CCL9/CCR1	CCL9 secreted by splenic macrophages induces a CCR1‑dependent accumulation of MDSCs in the spleen in a murine H22 hepatoma model	[49]
ENTPD2/CD39L1	MDSCs	Blockade of ENTPD2/CD39L1	Hypoxia induces the expression of ENTPD2 on cancer cells leading to elevated extracellular 5'-AMP, which promotes the maintenance of MDSCs by preventing their differentiation in HCC	[50]
PD-L1	MDSCs	PD-1 monoclonal antibody	PD-L1^+^ MDSCs could be used as a new biomarker of HCC	[51]
IL-18/TLR2	MDSCs	Blockade of IL-18/TLR2	IL-18 administration was sufficient to induce accumulation of MDSC, whereas hepatocyte-specific silencing of IL-18 in TLR2(-/-) mice decreased the proportion of MDSC	[52]
TGF-β/Axl/CXCL5	TANs	Blockade of TGF-β/Axl/CXCL5	The synergy of TGF-β and Axl induces CXCL5 secretion, causing the infiltration of neutrophils into HCC tissue.	[72]
cortisol	TANs	Inhibition of cortisol	increased cortisol production and TAN/TAM infiltration as primary factors in the gender disparity of HCC development in both fish and human	[73]
CXCR2/CXCL1	TANs	Blockade of CXCR2/CXCL1	The CXCR2-CXCL1 axis can regulate neutrophil infiltration into HCC tumor tissues and might represent a useful target for anti-HCC therapies	[74]
CXCL17	TANs	Anti-CXCL17	CXCL17 expression was associated with more CD68 and less CD4 cell infiltration	[75]
CXCR6	TANs	Anti-CXCR6	Human HCC samples expressing high levels of CXCR6 contained an increased number of CD66^+^ neutrophils and microvessels	[76]
miRNA-21	CAFs	MiRNA-21 inhibitor	High level of serum exosomal miRNA-21 was correlated with greater activation of CAFs and higher vessel density in HCC patients	[84]
CD24	CAFs	Anti-CD24	HGF and IL6 secreted by CAFs promoted the stemness properties of CD24^+^ HCC cells through the phosphorylation of STAT3	[85]
LOXL2	CAFs	Anti--LOXL2	The secreted LOXL2 promotes fibronectin production, MMP9 and CXCL12 expression and BMDCs recruitment to assist pre-metastatic niche formation	[86]
PD-L1/IL6/STAT3	CAFs	Blockade of PD-L1/IL6/STAT3	HCC-CAFs regulate the survival, activation, and function of neutrophils within HCC through an IL6-STAT3-PDL1 signaling cascade	[87]
IL6/STAT3	CAFs	blockade of IL6/STAT3	IL-6 secreted by CAFs promotes stem cell-like properties in HCC cells by enhancing STAT3/Notch signaling	[88]
Keratin 19	CAFs	Anti-Keratin 19	Keratin 19 expression in HCC is regulated by Fibroblast-Derived HGF via a MET-ERK1/2-AP1 and SP1 Axis.	[89]
LSD1	CAFs	Anti-LSD1	LSD1 Stimulates Cancer-Associated Fibroblasts to Drive Notch3-Dependent Self-Renewal of Liver Cancer Stem-like Cells	[90]
PD-1	Tregs	PD-1 monoclonal antibody	The ratio of CD4^+^CD127^+^ PD-1^-^ T effector cells to CD4^+^Foxp3^+^PD-1^+^ Tregs was significantly increased following treatment with sorafenib	[114]
PD-1	Tregs	PD-1 monoclonal antibody	Sunitinib-mediated tumoricidal effect and Treg suppression synergized with antibody-mediated blockade of PD-1 to powerfully suppress tumor growth and activate anti-tumor immunity	[115]
PD-1	Tregs	PD-1 monoclonal antibody	Sorafenib treatment enhanced functions of tumor-specific effector T cells as well as relieved PD-1-mediated intrinsic and Treg-mediated non-cell-autonomous inhibitions in tumor microenvironment	[116]
CTLA4	Tregs	CTLA4 monoclonal antibody	Leptin inhibited Treg activation and function in vitro, demonstrated by lower expression of TGF-β, IL-10, CTLA4 and GITR in Tregs	[117]
CTLA4	Tregs	CTLA4 monoclonal antibody	Tumor-induced regulatory DC subset suppresses antitumor immune response through CTLA-4-dependent IL-10 and IDO production	[118]
TIM3	Tregs	TIM3 monoclonal antibody	Antibodies against TIM3 restore responses of HCC-derived T cells to tumor antigens, and combinations of the antibodies have additive effects	[119]
Lnc-Tim3	Tregs	Anti-Lnc-Tim3	Lnc-Tim3 promotes T cell exhaustion, a phenotype which is correlated with compromised anti-tumor immunity	[120]
TIM3	Tregs	TIM3 monoclonal antibody	TIM3 -1516 G/T polymorphisms may affect the prognosis of HBV-related HCC and may be new predictors of prognosis for HCC patients	[121]
TIM3	Tregs	TIM3 monoclonal antibody	-1516G/T polymorphism in the promoter region of TIM3 gene may affect the disease susceptibility and HCC traits associated with HBV infection	[122]
GITR	Tregs	GITR monoclonal antibody	Agonistic targeting of GITR can enhance functionality of HCC TIL and may therefore be a promising strategy for single or combinatorial immunotherapy in HCC	[123]
GITR	Tregs	GITR monoclonal antibody	GITR-ligation and anti-CTLA-4 mAb can improve the antitumor immunity by abrogating Ti-Treg mediated suppression in HCC.	[124]
ICOS	Tregs	ICOS monoclonal antibody	Regulatory T cells, especially ICOS^+^ FOXP3^+^ regulatory T cells, are increased in the HCCmicroenvironment and predict reduced survival	[125]
OX40	Tregs	OX40 monoclonal antibody	OX40 expression in HCC is associated with a distinct immune microenvironment, specific mutation signature, and poor prognosis	[126]
LAG3	Tregs	LAG3 monoclonal antibody	Antibodies against LAG3 restore responses of HCC-derived T cells to tumor antigens, and combinations of the antibodies have additive effects	[119]

### Marrow-derived suppressor cells

**Fig. 2:**
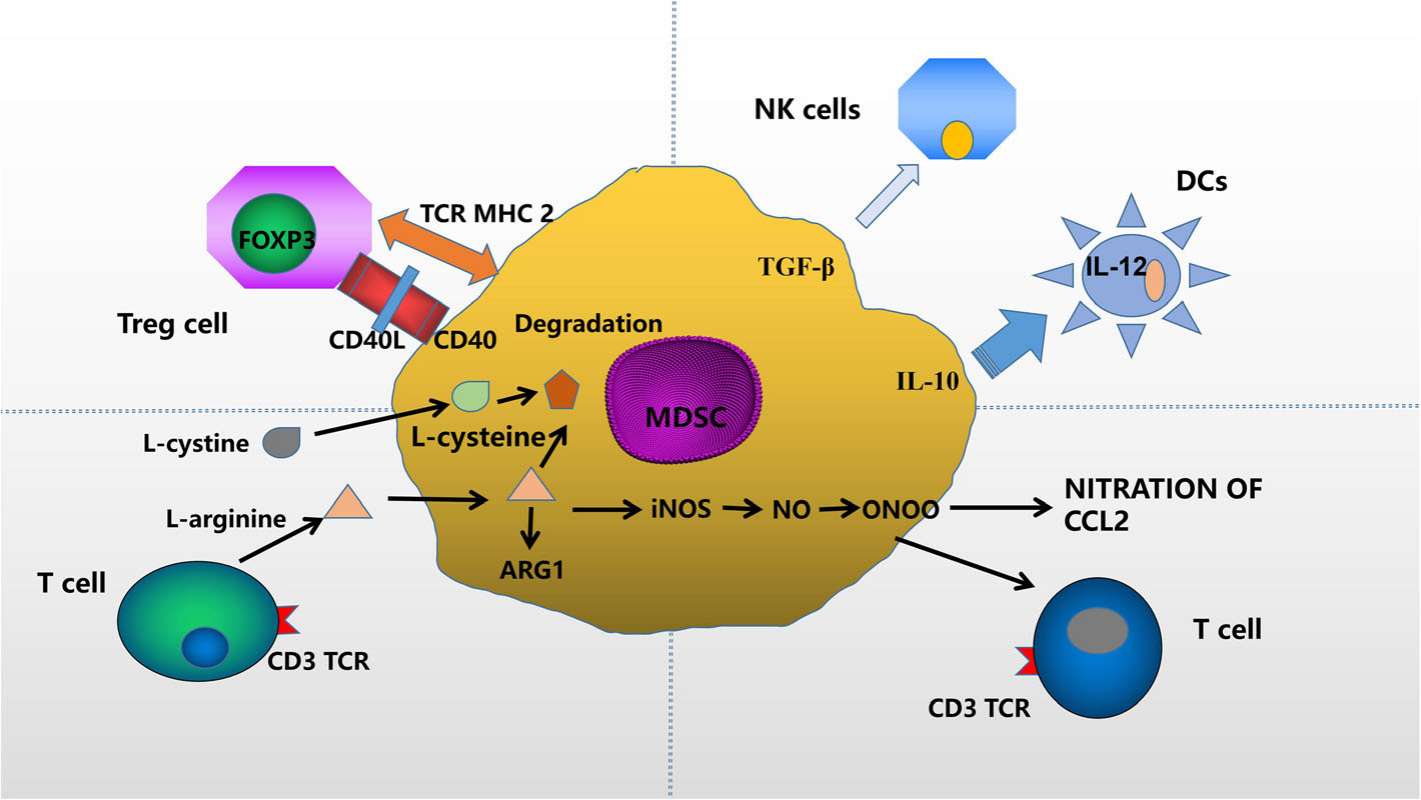
The mechanism of immunosuppressive activity of MDSCs in the tumor microenvironment. MDSCs induce differentiation and expansion of Tregs during tumorigenesis; inhibit DC, NK and macrophage polarization to the M2 phenotype; deprive T cells from essential amino acids; and produce oxidative stress to mediate cancer progress. Abbreviations:MDSCs: myeloid-derived suppressor cells; Treg cells: regulatory T cells;NK cells: natural killer cells; CCL2: CC-chemokine ligand 2; DCs: dendritic cell; FOXP3: forkhead box P3; IL-10: interleukin-10; iNOS: inducible nitric oxide synthase; TCR: T-cell receptors; TGF-β: transforming growth factor β

In cancer, the differentiation of myeloid cells often changes, producing a group of immature myeloid cells, which have strong immunosuppressive activity and impaired function as antigen-presenting cells (APCs) [39]. These cells are now known as MDSCs, a heterogeneous population of immature myeloid cell. MDSCs are also plastic and respond to microenvironment signals [40–42]. MDSCs can differentiate into macrophages, granulocytes and dendritic cells (DCs) in vitro. Therefore, MDSCs have significant diversity and plasticity, and are capable of changing their functional status in response to a variety of cytokines and growth factors in the tumor microenvironment. It has been reported that the immunosuppressive activity of MDSCs in the tumor microenvironment mainly includes (a) inducing differentiation and expansion of Tregs; (b) inhibiting the polarization of DCs, NKs and macrophages to the M2 phenotype; (c) depriving T cells of essential amino acids; (d) inducing oxidative stress (Fig. 2) [40–43]. We mainly reviewed the MDSCs in the tumor growth, angiogenesis, and metastasis of HCC in this context (Table 1) [44–52]. These evidence suggest that MDSCs contribute to the immunosuppressive network through multiple mechanisms and are potential immunotherapy targets for HCC.

MDSCs from HCC patients are unable to stimulate an allogeneic T-cell response, suppress T-cell proliferation, and have high arginase activity. Liu YT et al. used a hydrodynamic jet and transposon system to create a model that introduces a protein kinase and an open reading frames (ORFs) chromosomal encoding agent for tumor antigens. The transposon-based Akt/N-Ras-induced HCC mouse model allows researchers to monitor tumor growth non-invasively, quantify and characterize endogenous or over-transferred CD8^+^ T cell responses [53]. These characteristics make it a convenient preclinical model for the evaluation of immunological checkpoint inhibitors and cellular immunotherapy HCC. MDSCs was found to cause inhibition of CD8^+^ T-cell responses. In addition, recent studies have reported that inhibition of tumor CCRK or liver IL-6 increases interferon γ^+^ tumor necrosis factor-α^+^CD8^+^ T-cell infiltration and impaired tumorigenicity and is restored by recovery of MDSCs. It is worth noting that the tumor CCRK depletion up-regulated the expression of PD-L1 and increased the expression of intratumoral CD8^+^ T cells, thereby enhancing the effect of PD-L1 blocking HCC [54].

Additionally, these MDSCs cocultured with autologous T cells induce Treg expansion, which mitigates effector T-cell function. Kalathil S et al. recorded an increase in the number of Tregs, MDSC, PD-1^+^-exhausted T cells, and an increase in immunosuppressive cytokine levels in HCC patients, revealing the potential mechanistic network for immune disorders in HCC patients compared with the normal control group [55]. Other mechanisms have been described, in which MDSCs affect T-cell function, survival, and trafficking. Similar to TAMs, MDSCs express galectin-9 that binds to TIM-3 on T cells, inducing T-cell apoptosis [56]. MDSCs can also impair natural killer (NK) cell function. In HCC, MDSCs inhibit NK cell cytotoxicity and cytokine release, which is mediated by the NKp30 receptor [57]. Regarding the interaction between MDSCs and DC cells, it has been reported that MDSC inhibits TLR-ligand-induced IL-12 by IL-10 production and inhibits T-cell stimulating activity of DCs in HCC [58].

Because of the tumor-promoting and immunosuppressive effects of bone marrow cells, it is of great interest to target them to enhance the efficacy of conventional cancer therapies. The recently approved chemotherapeutic agent, trabectedin, not only targets tumor cells, but induces rapid apoptosis of bone marrow cells [59]. Clinical trials have shown that trabectedin also has a strong cytotoxic effect on liver cancer cells [60]. Therefore, trabectedin may be a potential treatment for HCC. Another potential target is estrogen, which inhibits myeloid cell function in HCC [61]. Estrogen inhibits IL-6 exposure to macrophages exposed to necrotic hepatocytes and reduces the risk of liver cancer in DEN-treated female mice [62]. Estrogen inhibits tumor necrotic cell-STAT6 activation by inhibiting Janus kinase, resulting in a reduced HCC model in mice [63]. Thus, estrogen therapy may help disrupt the development and function of HCC bone marrow cells. Therefore, targeting bone marrow cells represents a point of further research as a possible adjuvant therapy to attenuate HCC progression.

### Tumor-associated neutrophils

It is clear that bone marrow-derived cells, including TAMs, TANs, and MDSCs, promote tumor progression [64–66]. In recent years, many studies have shown that TANs not only promote tumor growth, but also anti-tumor effects on tumors, and can regulate their different phenotypes through tumor signal transduction. TANs can be divided into two major subtypes, N1 (anti-tumor) and N2 (pro-tumor) and the plasticity of these subtypes depends on the presence of TGF-β [67, 68]. Neutrophils can polarize TGF-β to the N2 phenotype while TGF-β together with increased inhibition of IFN-β induces N1phenotype [69].

Depending on the microenvironment of the tumor, TANs can promote or inhibit tumor progression by releasing cytokines. Zhou SL and his colleagues found that CCL2 and CCL17 are the most highly expressed cytokines in peripheral blood neutrophils (PBNs) activated by TANs and HCC cells. The number of CCL2^+^ or CCL17^+^ TANs is related to tumor size, microvascular infiltration, tumor embedding, tumor differentiation and staging. Compared to PBN-conditioned medium, TAN-conditioned media and recombinant CCL2 and CCL17 increased the migration activity of HCC cells or mouse macrophages and Tregs [70]. Taken together, this evidence suggests that TANs recruit macrophages and Treg cells into HCCs to promote their growth, progression, and resistance to sorafenib. Surprisingly, the same team reported that the secretion secreted BMP2 and TGF-β2 and triggered the expression of miR-301b-3p in HCC cells, followed by inhibition of the limbal gene expression membrane protein (LSAMP) and CYLD Lysine deubiquitinase and increased stem cell characteristics in HCC cells. These TAN-induced hepatoma stem cell-like cells are active in NF-κB signaling, a higher level of secretion of C-X-C-thematic chemokine 5 (CXCL5) and recruitment of more TANs infiltration, suggesting a positive feedback loop [71]. Additionally, we have literature some important molecules which might target TANs in HCC (Table 1) [72–76].

### Cancer-associated fibroblasts

Recent studies have shown that communication between cancer cells and fibroblasts is very important, and these fibroblasts are called CAFs [77, 78]. CAFs promote the proliferation, invasion and metastasis of tumors by secreting various growth factors and cytokines [79, 80]. In HCC, Mano Y et al. established that primary CAFs and non-cancerous liver fibroblasts from 15 patients undergoing HCC resection. They found that endogenous and exogenous BMP4 activate hepatic fibroblasts to gain the ability to secrete cytokines and enhance the invasiveness of cancer cells. BMP4 is one of the regulators of CAFs function in the HCC microenvironment [81].

CAFs train NK cells to obtain an inactive phenotype and produce an unresponsive state in the tumor. Li T et al. found that HCC-derived fibroblasts induced NK cell dysfunction significantly better than foreskin-derived fibroblasts, which were characterized by low expression of cytotoxic molecules and cell surface markers, and impaired production of cytokines. They also noted that PGE2 and IDO from activated fibroblasts inhibit the activation of NK cells, thereby creating favorable conditions for tumor progression [82]. In addition, CAF can recruit regulatory DCs through IL-6-mediated STAT3 activation and educate them to obtain a tolerogenic phenotype and up-regulate Treg production by secreting TGF-β in the tumor microenvironment [83]. In summary, CAFs play an important role in the development and progression of cancer cells and targeting CAFs may be effective in treating fibrosis and preventing HCC progression (Tables 1, [84–90].

Anticancer therapies targeting CAFs or cytokine inhibitors secreted by CAFs have recently been actively studied. Inhibitors of TGF-beta signaling have been shown to block HCC growth and progression by modulating EMT in different experimental models [91]. Several studies have explored the active targeting of CAFs to provide therapeutic compounds. A recent report presented anti-fibrotic drugs such as PRI-724, conophylline, follistatin, salvianolic acid, Gliotoxin. Curcumin, sulfasalazine, and tanshinone I, which inhibit activated HSC and/or induce apoptosis [92–96]. It is believed that these drugs can not only control fibrosis, but also inhibit HCC by controlling the function of CAFs. It has been reported that fibroblast growth factor receptor 1 is overexpressed in the fiber layer HCC [97]. CAFs stimulate tumor cells through FGF and produces fibrosis [98, 99]. Treatments that target CAFs may be effective in fibrolamellar HCC. CAFs are one of the most important components in the tumor microenvironment and promote cancer cell growth and invasion through various mechanisms. CAFs are important for the initiation and progression of cancer cells and for the treatment of CAFs to effectively treat fibrosis and prevent HCC progression.

### Tumor-infiltrating Tregs

Tregs cells are the subset of CD4^+^ T cells and identified with the CD25 marker. CD4^+^ CD25^+^ compartment of cells is approximately 1–2% of peripheral CD4^+^cells. Tregs cells do not only limit autoimmune responses, they also dampen responses against microbial and viral antigens, allergens, tumors and allografts. Tumor-associated Tregs directly promote tumor evasion by a number of contact-dependent and contact-independent mechanisms [100, 101].

The number of Tregs has been reported to increase in patients with HCC [102]. Yang et al. found that the proportion and absolute number of CD4^+^CD25^+^ T cells in the surrounding area of the tumor were significantly increased [103]. The other group showed a higher frequency of Tregs in peripheral blood from HCC patients compared to HCV patients and healthy subjects. Mechanistically, Huh7 culture supernatants appear to promote CD4^+^CD25^+^ T-cell proliferation and inhibit CD4^+^CD25^−^ T-cell proliferation [104]. In addition, the frequency of circulating Tregs was linked to the disease progression and had a potential of serving as a significant biomarker in HCC patients [105, 106]. Sorafenib is a multikinase inhibitor that could reduce the frequency of hepatic infiltrating Tregs by suppressing TGF-β signaling [107]. As a result,these evidence indicates suppressing Tregs might be one of the significant targets for the induction of an immune response for HCC.

**Fig. 3:**
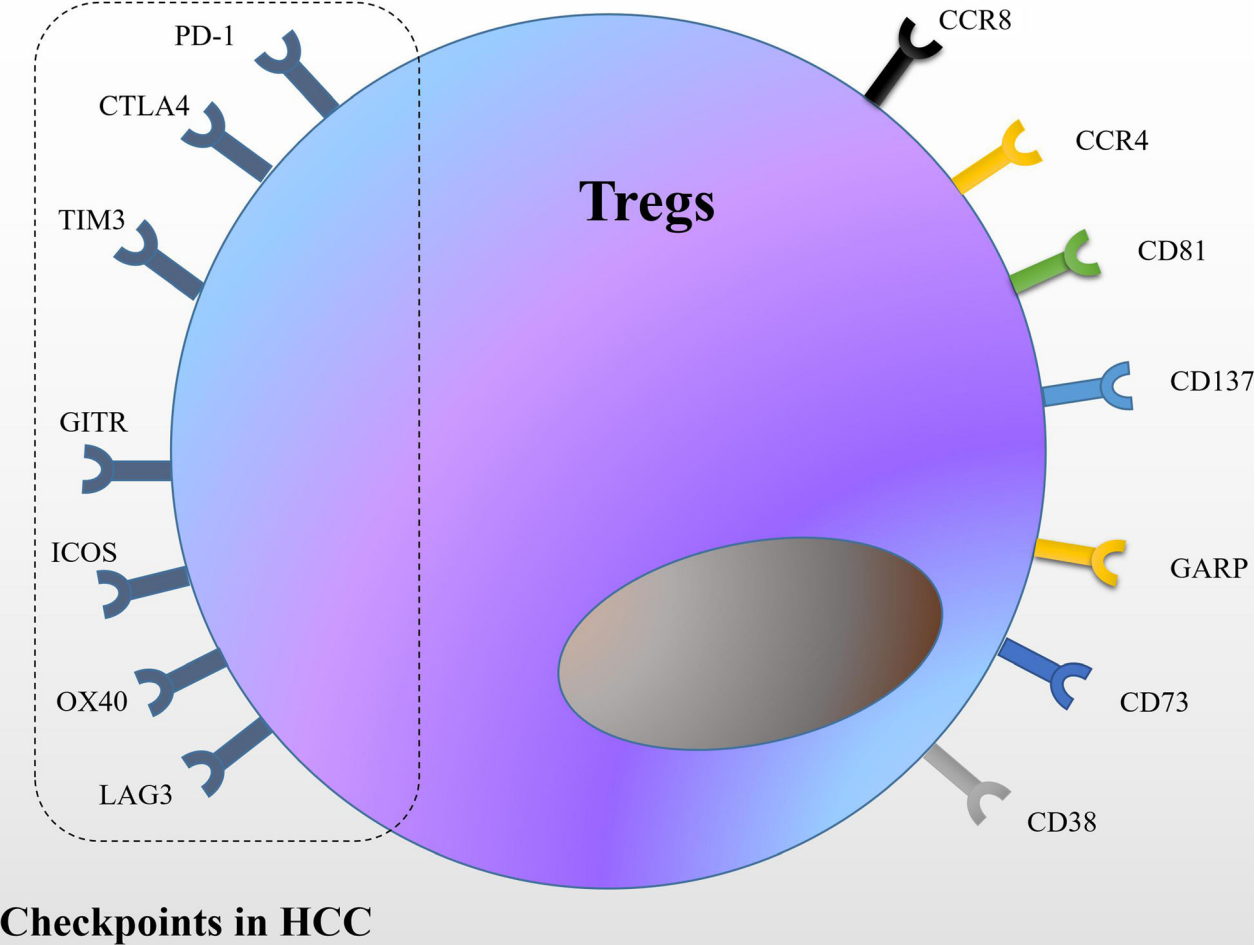
Summary of potential candidates for Treg-targeted anti-tumor immunotherapy in HCC. Targets such as CCR4, PD-1, LAG3, TIM3 and GITR are primarily expressed on the membrane surface of Treg cells. Checkpoint inhibitors overcome T cell failure in HCC progression and restore the immunosuppressive state of the HCC microenvironment by blocking immunological checkpoint molecules. Abbreviations:CTLA4:cytotoxic T-lymphocyte-associated protein 4;PD-1:programmed cell death 1; TIM3: T cell immunoglobulin domain and mucin domain-3;LAG3: lymphocyte-activation gene 3;GITR:tumor necrosis factor receptor superfamily, member 18;ICOS: inducible T-cell co-stimulator;OX40: tumor necrosis factor receptor superfamily, member 4;CCR8:chemokine (C-C motif) receptor 8;CCR4:chemokine (C-C motif) receptor 4;GARP:glycoprotein-A repetitions predominant

CTLA-4 is member of immunoglobulin superfamily homologous to CD28 with higher affinity for B7. CTLA-4 inhibits early T cell immune response, mainly in lymphoid tissues [108]. In naive T cells, CTLA-4 localized in the intracellular space and expressed on the cell surface upon stimulatory signals. Whereas in Tregs, CTLA-4 is constitutively expressed and is involved in Treg suppressive functions [109]. Several studies investigated PD-1 and PD-L1 expression in the context of HCC and found that PD-L1 expression was localized mainly in neoplastic or intratumoral inflammatory cells [110, 111]. Moreover, PD-1/PD-L1 interaction was demonstrated to contribute to immune suppression in HCC [112]. High level of PD-L1 expression was shown in tumors with a high number of tumor infiltrating lymphocytes (TILs) and shorter overall survival [113]. There are several clinical trials investigating the efficacy of monoclonal antibodies (mAb) against CTLA-4 and PD-1/PD-L1 as single agent treatment. In this review, we have extensively studied the efficacy of the current proposed immunotherapy for Tregs in patients with HCC. (Fig. 3) (Tables 1,2) [114–127].

**Table 2:** Clinical trials in HCC using immune checkpoint inhibitors

Antibody features	Antibody name	NCT number	Status
anti-CTLA4	Ipilimumab	NCT03682276,NCT03510871,NCT03222076,NCT03203304	Ongoing
anti-CTLA4	Tremelimumab	NCT01853618,NCT02519348,NCT02519348,NCT03298451, NCT02821754,NCT03482102, NCT03638141	Ongoing
		NCT01008358	Completed
anti-PD-1	Nivolumab	NCT01658878,NCT02576509,NCT03383458, NCT03682276, NCT03071094,NCT03382886, NCT03781960, NCT03033446, NCT03510871, NCT03299946, NCT03059147,NCT03630640, NCT03418922, NCT03655613,NCT02837029, NCT03785210, NCT03695250,NCT03655002, NCT02859324, NCT03439891	Ongoing
anti-PD-1	Pembrolizumab	NCT02702401,NCT03062358,NCT03713593,NCT02658019, NCT03753659,NCT03006926,NCT03519997,NCT03337841, NCT03163992,NCT03397654,NCT03316872,NCT03099564, NCT02940496,NCT03419481,NCT03647163,NCT02509507, NCT03211416,NCT03347292,NCT03511222,NCT03095781, NCT03259867,NCT02595866, NCT02432963	Ongoing
anti-PD-1	Tislelizumab	NCT03419897, NCT03412773	Ongoing
anti-PD-1	Spartalizumab	NCT02988440,NCT02795429, NCT02947165,NCT02325739	Ongoing
anti-PD-1	Camrelizumab	NCT02989922,NCT03463876,NCT02942329,NCT03722875, NCT03605706, NCT03793725,NCT03764293, NCT03601598	Ongoing
anti-PD-L1	Durvalumab	NCT03298451,NCT03778957,NCT03482102,NCT02519348 NCT03638141,NCT03257761,NCT02821754,NCT02572687, NCT03539822	Ongoing
anti-PD-L1	Avelumab	NCT03389126, NCT03289533	Ongoing
anti-PD-L1	Atezolizuamb	NCT03170960,NCT03755791, NCT03434379	Ongoing

## Conclusion and perspectives

Due to high mortality, limited treatment and poor prognosis of HCC, new immunotherapy treatments are urgently needed. For example, blocking the activation of immunosuppressive receptors on T-cells has become a new focus of therapy. Immunosuppressive cells, including TAMs, MDSCs, TANs, CAFs, and Tregs are key components of the tumor microenvironment that promote HCC growth and invasion. There is an interaction between these types of immune cells leading to tumor immune escape. These cells often have both anti-cancer and cancer-promoting effects, and their specific regulation and mechanism of action are not well understood. The differentiation, maturation and function of immune cells require the participation and regulation of cytokines and chemical factors, as well as the interaction of receptors and related ligands. These factors create a tumour microenviroment that inhibits the anti-tumor activity of immune cells, promoting the occurrence of HCC. Further research involving paired tumor biopsy will inform immunotherapy treatments and improve the prognosis of patients with HCC. In addition, advances in DNA and RNA sequencing technologies provide insights into the mechanisms of HCC progression to identify other targets. Altogether, these approaches to treatment brings new hope to HCC patients.

## Data Availability

Not applicable.

## Abbreviations

AMD-NPs: AMD-modified NPs
APCs: Antigen-Presenting Cells
CAFs: Cancer-associated fibroblasts
CSF: Colony stimulating factor-1 receptor
CTLA-4: Cytotoxic T lymphocyte-associated protein 4
CXCL5: C-X-C-thematic chemokine 5
DCs: Dendritic Cells
HCC: Hepatocellular carcinoma
IL-10: Interleukin-10
LSAMP: Limbal gene expression membrane protein
mAb: Monoclonal antibodies
MDSCs: Marrow-derived suppressor cells
MMPs: Matrix metalloproteinase
NK: Natural killer
NP: Nanoparticles
ORFs: Open Reading Frames
PBNs: Peripheral blood neutrophils
PD-1: Programmed cell death protein 1
PD-L1: Programmed cell death ligand 1
TAMs: Tumor-associated macrophages
TANs: Tumor-associated neutrophils
TILs: Tumor infiltrating lymphocytes
TLR2: Toll-like Receptor 2
Tregs: Regulatory T cells
VEGF: Vascular Endothelial Growth Factor
ZA: Zoledronic Acid
